# Unveiling Spatial Epidemiology of HIV with Mobile Phone Data

**DOI:** 10.1038/srep19342

**Published:** 2016-01-13

**Authors:** Sanja Brdar, Katarina Gavrić, Dubravko Ćulibrk, Vladimir Crnojević

**Affiliations:** 1 Faculty of Technical Sciences, University of Novi Sad, Novi Sad, 21000, Serbia; 2 Department of Information Engineering and Computer Science, University of Trento, Trento, 38122, Italy; 3 BioSense Institute, University of Novi Sad, 21000, Serbia

## Abstract

An increasing amount of geo-referenced mobile phone data enables the identification of behavioral patterns, habits and movements of people. With this data, we can extract the knowledge potentially useful for many applications including the one tackled in this study - understanding spatial variation of epidemics. We explored the datasets collected by a cell phone service provider and linked them to spatial HIV prevalence rates estimated from publicly available surveys. For that purpose, 224 features were extracted from mobility and connectivity traces and related to the level of HIV epidemic in 50 Ivory Coast departments. By means of regression models, we evaluated predictive ability of extracted features. Several models predicted HIV prevalence that are highly correlated (>0.7) with actual values. Through contribution analysis we identified key elements that correlate with the rate of infections and could serve as a proxy for epidemic monitoring. Our findings indicate that night connectivity and activity, spatial area covered by users and overall migrations are strongly linked to HIV. By visualizing the communication and mobility flows, we strived to explain the spatial structure of epidemics. We discovered that strong ties and hubs in communication and mobility align with HIV hot spots.

HIV has a devastating social, demographic, and economic effect on Africa^1^,^2^. With a 3.7% of population infected^3^, the Ivory Coast has the highest prevalence rate in West Africa and is generalized as an epidemic^4^,^5^. The disease spreads out of the risk groups and affects entire population, demanding the development of national HIV-prevention plans. The prevalence rate appears to be relatively stable over the past decade (even decreasing), due to prevention of mother-to-child transmission. Still, there is a lot of work to be done in order to improve the health system and to enable a more effective response to HIV. Deeper understanding of the epidemics can help find the ways to suppress HIV further and modern technologies that deal with human mobility phenomena may help respond to that challenge.

Mobile phone communication engendered the era of big data by creating huge amounts of Call Detail Records (CDRs). Cell phone service providers collect these records whenever a phone is used to send a text message or make a call. These records contain the time of the action, identifiers (IDs) of sender, receiver and the cell towers used to communicate. In this way, mobile phones provide approximate spatio-temporal localization of users and create an immense resource for the analysis of human mobility and behavioral patterns^6^,^7^,^8^. With a exponential growth of new applications built on mobile phone data^9^, there are those of a great practical importance such as: urban planning^10^, disaster management^11^, transportation mode inference^12^, traffic engineering^13^, deriving poverty indicators^14^ and crime prediction^15^.

Currently, there is a growing interest in the mining of mobile phone data for epidemiological purposes^16^,^17^. Mining this data can advance research in epidemiology by shedding light on the relationships between disease distribution, spread and incidence on one side, and migrations, everyday movements and connectivity of people on the other side. Up to now, only a few studies have used mobile phone data to quantify those relationships based on real disease distribution data. Wesolowski and co-workers explored the impact of the human mobility to the spread of malaria^18^. They analyzed CDR data collected by a mobile phone service provider in Kenya over the period of one year and discovered how human mobility patterns contribute to the spread of the disease beyond what could be possible if it was transferred only by insects. Another study carried by Martinez *et al.*^19^ investigated the effect of government alerts during H1N1 flu outbreak in Mexico on the diameter of the mobility of individuals. Bengtsson and co-workers^11^ estimated population movements from a cholera outbreak area and suggested the use of information obtained for disease surveillance and resolving priority in relief assistance. These pioneering works usher in the emerging field of digital epidemiology^20^.

To the best of our knowledge, the study we describe here is the first attempt to use mobile phone data to explore the complex structure of HIV epidemics. Significant scientific effort is aimed at identifying the driving factors of HIV spread. Most frequently mentioned are poverty, social instability, violence, high mobility, and rapid urbanization and modernization. The differences among these factors could help explain the spatial disparity observed in prevalence rates. Messina *et al.* examined geographic patterns of HIV prevalence in Democratic Republic of Congo^21^. They showed that spatial factors: the prevalence level in the 25 km range and the distance to the urban areas are strongly connected to the risk of HIV infection. The impact of migration on the spread of HIV in South Africa has been studied in^22^, where the authors developed a mathematical model to compare the effects of migration and associated risk behavior. In the early stage of epidemics, migration impacts the HIV progression by linking geographical areas of low and high risk. In the later stage, the impact is mainly through the increase the high risk sexual behavior. The migration in the study was quantified through surveys. In these surveys, the participants were questioned about movement history, and included only two migration destinations, limiting both the extent of usage of study, and the quality of data that was used.

Today, the large amounts of mobile phone data records provide us insight into the movements and activity of millions of people over large areas, which can be utilized for new studies of the epidemiology of HIV. In the study described here, we conducted a comprehensive analysis of two data sets offered within the Data for Development (D4D) Challenge^23^. Our research was guided by the following hypothesis: the risk of HIV infection is associated with spatial and behavioral factors that can be detected from the collection of data available. We were particularly interested in tracking population movements and inferring the strength of communication between departments of the Ivory Coast with different prevalence rates.

## Results

### Spatial distribution of HIV

**Figure 1:**
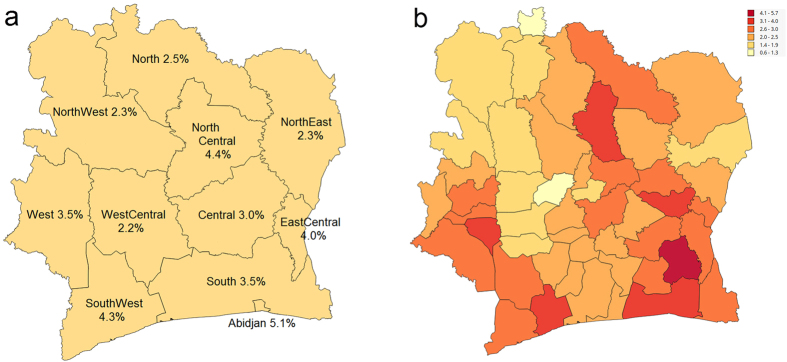
(**a**) HIV prevalence rate by administrative regions (**b**) HIV prevalence rate by departments for 15–49 year-olds population; estimated values range between 0.6 and 5.7%. (We used open source QGIS software^59^ to create maps from (**a**) DHS data^3^ (**b**) UNAIDS estimates^25^).

To determine the health status of a population, Demographic and Health Surveys (DHS) periodically organizes surveys to gather relevant data, focusing on specific countries. In our study we used the DHS data collected in the Ivory Coast during their 2012 campaign^3^. Based on the measurement, DHS provides estimates of HIV prevalence at sub-national level with a low spatial resolution, determined by 10 administrative regions (Fig. 1(a)). Estimates of the HIV prevalence rate range from 2.2 to 5.1% and reveal the spatial variability of the distribution of HIV infection across the country.

Due to initiatives to examine the spatial heterogeneity of HIV^24^, new methods emerged, aiming to provide HIV estimates at a finer resolution. An approach that employs kernel estimation based on spatial DHS measurements with an additional adjustment to UNAIDS data, made estimates for 50 departments of the Ivory Coast available^25^ (see Methods). After redistributing disease frequencies across 50 departments, the HIV prevalence map (Fig. 1(b)) shows higher spatial variability in disease distribution from 0.6 to 5.7%. We can notice the hot spots of epidemics – departments severely hit by HIV. The map enables us to explore links between the connectivity and mobility patterns derived from D4D data and HIV prevalence with increased spatial resolution. Although the quality of HIV estimates (imposed by DHS measurement sampling) at department level varies from good and moderate to uncertain, the data has the highest spatial resolution currently available for studying the HIV epidemic in the Ivory Coast.

**Figure 2:**
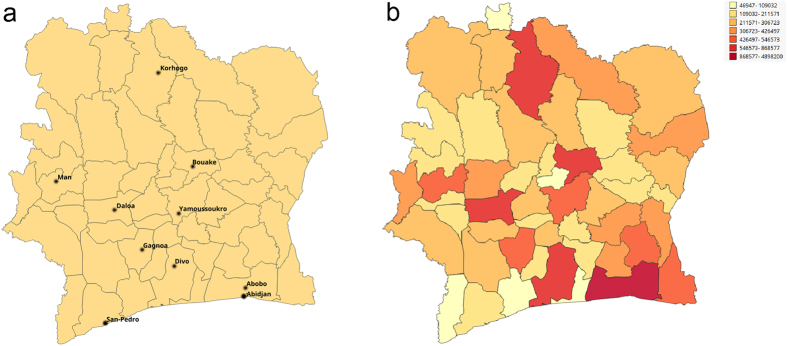
(**a**) 10 largest cities of the Ivory Coast (**b**) Population distribution across departments. (We used open source QGIS software^59^ to create maps).

Along with HIV distribution map, we provide additional maps showing locations of the 10 largest cities of the Ivory Coast (Fig. 2(a)) and population density aggregated at the departments level (Fig. 2(b)), that are necessary for understanding of the results.

### Communication and mobility patterns

Social interactions and mobility mediate the spread of infectious diseases^17^,^26^,^27^. When examined in a spatio-temporal context, they can uncover how a disease propagates and can explain the variability in the prevalence distribution. Epidemic patterns can be studied at different scales spanning from short range commuting flows to the long range intercontinental connections^28^. The level of detail in quantifying social interactions and mobilities can be chosen according to the scale of interest. While global epidemics patterns are mainly determined by the airline network, for country level epidemic we need finer resolution data sources. To better understand spatial epidemiology of HIV across 50 departments of the Ivory Coast, we analyzed the collective communication and mobility connections from mobile phone data. We estimated pairwise connections among departments by measuring communication and mobility flows. To accomplish that, we explored the “antenna-to-antenna data” (SET1) and the “long term individual trajectories” (SET3) in D4D dataset^23^.

SET1 provided us with insight into the communication flow between each pair of antennas on an hourly basis. The strength of the communication flow is expressed through the number of calls. We assigned each antenna to a corresponding department and then aggregated the number of calls at the department level during a 5-month observation period. SET3 shed light on the mobility of people, providing the geographic location of users while using their phone to make calls or send messages. Since records in SET3 contain the user ID, location at the sub-prefecture level and time stamps indicating when the phone was used, we were able to use them to estimate the location of the user’s home. Based on the most frequent location, we assigned each user to their home department. Then we counted the user’s movements from home to other locations over the entire 5-month observation period and aggregated users’ movements at the department level.

In the pairwise communication and mobility matrices, we identified *strong ties* for each department, which represent links to other departments with the connection strength significant at *α* = 0.01 (see Methods). Before searching for the strong ties, we normalized the matrices by the corresponding population sizes. SET1 encompasses 5 million of users. We distributed them into departments, using population frequencies provided by Afripop data^29^, and used the per-department populations obtained to normalize the communication flows. To normalize the migration flows, we used estimates based on the derived home locations of the users to calculate the required population size per department. Each communication or mobility flow was normalized by the corresponding population size of originating department. The overall flow between two departments was then quantified as sum of normalized flows in both directions. This enabled us to eliminate the bias caused by the different population sizes when identifying the strong links.

**Figure 3:**
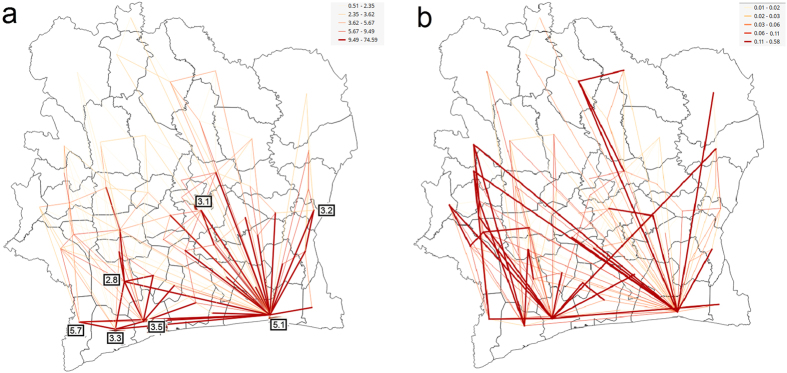
Strong connectivity ties for (a) overall communication (b) night communication. The hubs are labeled with the corresponding HIV prevalence rate shown in Fig. 1(b). Link thickness and color, ranging from yellow to red, are proportional to the strength of communication flow. (We used open source QGIS software^59^ to create maps).

The strong ties discovered in communication flows are shown in Fig. 3(a). This visualization emphasizes the strongest links further and communication hubs emerge. Remarkably, the hubs correspond to HIV hot spots and we can also notice that larger hubs have higher prevalence rates. The map at Fig. 2(a) helps us to reveal how identified hubs correlate with locations of urban centers. The largest hub corresponds to the department with two largest cities Abidjan and Abobo and it has degree of 46 significant links. The other highly connected hubs are located in the Southwest region in departments with IDs 38, 37, 42 (see Fig. S1) with degrees 22, 12, and 8 and all are severely hit by HIV. What is interesting is that departments 38 and 42 do not contain any of the top 10 cities. San Pedro, that is the fifth ranked is located in the department 37. There are also hubs around the cities Yamoussoukro and Bouake. We can notice that communication hubs usually correspond to the departments with large urban centers, but not necessarily, as we have also observed hubs without large cities.

Additionally, we visualized the night communication, constrained to the time interval between 1 AM and 5 AM, and obtained a similar structure of the connectivity graph - Fig. 3(b). The links of night communication are colored with the same palette as overall communication, but relatively to theirs maximum. Values of absolute flows are available in the legend. The largest hub is around Abidjan that has degree of 49. The size of hubs at the Southwest region additionally increased.

In both graphs we can notice how departments in the north part of the country have weaker links. The link’s strength, quantified as normalized communication flow between departments, includes both - residents of the departments and visitors. In this context, weaker links imply less social interactions or lower department’s attractiveness for visitors, or interplay of both. As social connections shape movement patterns and increase likelihood of contact between individuals^30^, presented graphs could help in understanding disease spatial distribution. Visually apparent sparser and weaker social connectivity in the north part of the country may have affected epidemic spread by making it harder for disease to propagate. This potentially explains smaller HIV prevalence in the north of the Ivory Coast.

**Figure 4:**
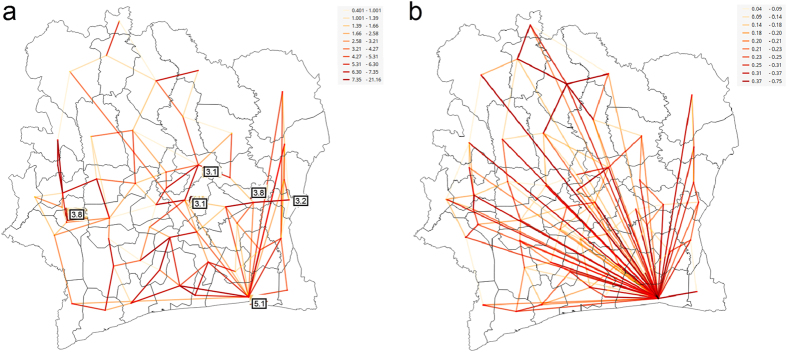
Strong mobility ties discovered through summarizing (a) all mobilities (b) mobilities with 3 days or longer stay at the destination. The hubs are labeled with the corresponding HIV prevalence rates shown in Fig. 1(b). The link thickness and color, ranging from yellow to red, are proportional to the strength of mobility flow. (We used open source QGIS software^59^ to create maps).

The strong ties discovered in mobility flows (Fig. 4(a)) have an obvious localized character. They connect the departments that are geographically close, but, on a global scale, we can also observe strong migratory pathways. One connects the two largest hubs - the largest city Abidjan (5.1% prevalence rate) and the capital city Yamoussoukro (3.1% prevalence rate). From the center of country we can notice strong pathways to the region in the West (3.6% prevalence rate, Fig. 1(a)) and the North-central region (4.0% prevalence rate, Fig. 1(a)). The East-central region, with a prevalence rate of 4.0% is strongly connected to Abidjan. The map of the mobility flows revealed the pathways that connect regions with higher prevalence.

In addition to the observed general mobility of users, we explored the long-term mobility. We measured how long users stay at their destinations and in our migration analysis considered only those stays in which the users stayed longer than 3 days. The strong ties discovered in long-term mobility flows are shown in Fig. 4(b). The connectivity graph obtained, reveals how long-term migrations link departments. Abidjan emerged as the most prominent hub for those migrations, with the hub degree of 49. In this light, we can denote this city, with the largest prevalence rate and high connectivity, as a driver of epidemic in the Ivory Coast. As such, Abidjan needs careful monitoring of mobility flows, especially the high-risk longer-term mobilities, in order to prioritize interventions and control the further spread of HIV.

### Extracted features

For each department of the Ivory Coast, numerous features were extracted during the course of the study presented, with the goal to quantify behavioral and mobility patterns potentially relevant to the measured HIV prevalence rate. Overall, we extracted 224 different features and grouped them into 4 categories: connectivity, spatial, migration and activity (phone use).

The connectivity features were obtained from the SET1. The communication flow is expressed through the number of calls and their duration in SET1. For each department we used the information on the originating and terminating antenna, and aggregated its inner, originating, terminating and overall communication. The overall communication was further separated based on the type of day and time of day constraints. We considered two types of days: weekdays and weekends, and used 1-hour time slots (00–01 h, 01–02 h, …, 23–24 h) and 8-hour time slots (00–08 h, 08–16 h, 16–24 h) to express the time within a day. For each of these discrete intervals, the features related to the number of calls represent the sum over the whole five-month observation period. Once extracted they were normalized by the corresponding department population size, estimated based on Afripop data^29^ and rescaled to fit the 5 million of users monitored in our data set. Features related to the duration of calls represent average values. 120 connectivity features related to different time slots and type of days were extracted; half to describe the number of calls and half to describe the average duration of calls.

Spatial, migration and activity features were derived from SET3. To craft spatial features we explored positions and the distribution of locations visited by users. We measured the radius of gyration, area and the perimeter of convex hull of users’ movements, as well as the diameter of their range^31^,^32^,^33^. The features were derived both for all locations visited by a user, as well as specific subsets of locations: visited at night, on weekdays, weekends, weekday and weekend nights. In addition, we calculated the total distance travelled by each user. In total, 25 spatial features were created, representing 95 percentile values across users matched to departments based on their home location. We first considered averaged instead of 95 percentile values for users in corresponding departments, but for predictive models better results are achieved when spatial features capture only the top five percent of users; i.e. the patterns of users that cover larger regions through their mobility have higher predictive power on the prevalence of HIV.

To extract migration features we tracked the changes in locations. Every time a user changed department, we added a single migration link from his home to the observed department. We summarized all movements into a pairwise migration matrix by iterating this procedure for all users. Beside quantifying all movements, we also identified those where users were away from home for more than defined number of days (1, 2, …, 10) to explore longer-term migrations. The features were divided further according to the direction of the mobility into “in” or “out” migration, bringing total number to 22.

The activity features were extracted similarly to the connectivity features. However, in SET3, we cannot distinguish the direction of communication (in or out), nor do we have the duration of communication. Therefore, we refer to those features simply as activity since they can count only when and where users were active. As with the connectivity features we considered two types of days: weekdays and weekends. The time of day was again considered in 1-hour time slots, 8-hour time slots and whole days. The total number of activity features used was 57.

All the features capture the cumulative effect of human connectivity or mobility observed over a five-month period. We focused on this long-term perspective in our feature extraction, in order to understand the spatial distribution of HIV prevalence better.

### Predictive models

HIV prevalence rates across the departments of the Ivory Coast range from 0.6 to 5.7%. Each of the 50 departments was represented with a vector of extracted features values and the corresponding prevalence rate. In this feature space, we built regression models and evaluated their performance when predicting a department’s prevalence rate. All features were normalized by dividing each feature with its mean value across the whole data set, before regression was attempted.

Experiments were conducted using two different regression methods: Ridge^34^ and Support Vector Regression (SVR)^35^. The regression models were initially built using the four different groups of features separately. In order to select smaller subsets of most relevant features, both regression methods were subjected to recursive feature elimination RFE^36^ method. In the final stage, we considered an ensemble approach – stacked regression^37^, through which we fused 4 heterogeneous feature sets, building a single integrated prediction model.

The prediction of disease levels needs careful evaluation^38^ in order to avoid situations in which models built on randomly generated data work comparatively well to those created on possibly meaningful data. Therefore, to estimate the predictive capacity of a model, we measured the prediction errors and correlations between the predicted and actual values for the models built on real data and the same models created based on random data sets, obtained by randomly permuting values for each feature.

Experiments were divided into two parts: the first stage focused on the 15 departments with good and moderate estimates of HIV prevalence, while in the second we used data for all 50 departments. In Tables 1 and 2, we report the correlation coefficients (*ρ*) and relative root mean square errors (*RRMSE*) produced by the models during leave-one-out (LOO) cross-validation for two experimental setups (15 and 50 departments).

Leave-one-out (LOO) evaluation enabled us to select the best model among those we built. On the subsample of 15 departments, the models built with SVR, with Recursive Feature Elimination (RFE), perform best. In the best models RFE reduced the initial set of features at subset of 60, 6, 3, 4 for connectivity, spatial, migration and activity features, respectively. Selected features are highlighted in Tables S2 and S3 that includes all features and their descriptions. SVR models surpassed Ridge and reducing the size of the feature set with RFE improved performance of both, but the SVR method benefited more from the RFE procedure than Ridge. The highest correlation coefficient (0.753) between the predicted and actual values is achieved with the SVR on a reduced set of 6 most relevant spatial features. The lowest error of 0.287 is reached by combining regression learned on different sets of features. Through the linear combination of the four models, the ensemble approach predicts HIV prevalence values that are well correlated with actual (*ρ* = 0.710). All models built on the real features outperformed their random counterparts.

The second part of the experiments evaluated the proposed methods and extracted features on the full set of 50 departments, including those with uncertain estimates on HIV. Table 2 reports the obtained results. As expected, the performance declined. Predictions are moderately correlated with actual values. The best result *ρ* = 0.627, *RRMS* = 0.509 is achieved with the SVR model on a reduced subset of activity features. Ensemble approach that combines four SVR + RFE models results in *ρ* = 0.518 and *RRMSE* = 0.514. The models created on randomly permuted features predict HIV with higher errors and without correlation with actual values and underperform those built on real features.

### Feature contribution

Once a regression model is built, we can use it to estimate the risk of disease in defined spatial units. Furthermore, we can examine what the model learned from the data. Model explanation techniques^39^,^40^ can unveil black-box predictive models by estimating contributions of each feature over the whole range of its input values. For example, we can examine how changes in an activity feature affect the value of the HIV prevalence rate, obtained by the model built. The outcome is a plot of the contribution as a function of feature values. This model-explanation procedure provides us with the opportunity to identify specific features that impact prevalence rate most of all and to quantify their contribution. The features identified in this manner can later be continuously measured and leveraged for the monitoring of changes in the HIV prevalence rate and to create early warning signs for possible increase of the infected population.

**Figure 5:**
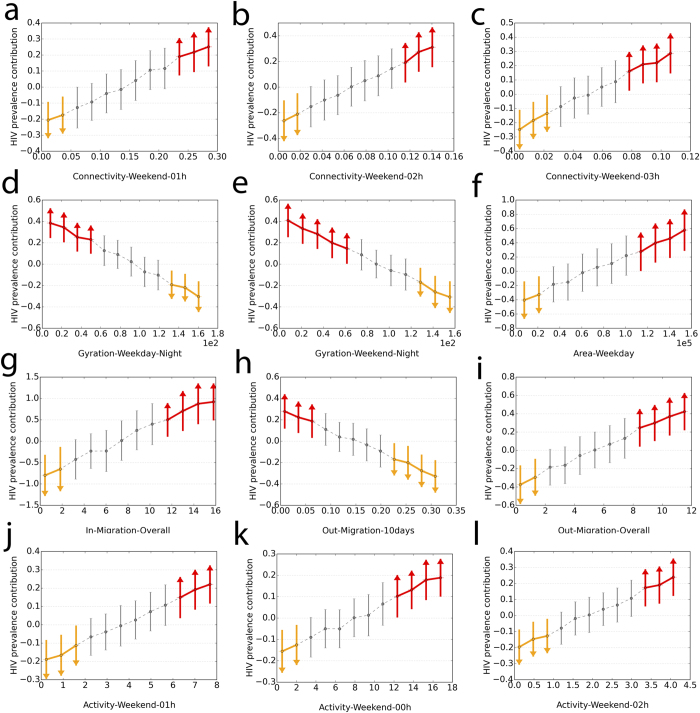
Feature contribution graphs for 12 features; top 3 features for 4 types of features. Points correspond to the mean contribution and error bars correspond to standard deviation. Red color indicates strong association to higher HIV prevalence, and orange to lower HIV prevalence.

To conduct the feature contribution analysis, we used the best model (SVR + RFE) built for each set of features, since the ensemble method is just an additive combination of models built on different sets. In the analysis we used models built on a subsample of departments (15 with good or moderate HIV estimation) and focused on the highly ranked features. By running the RFE procedure until only one features remains, we obtained ranks for all features and then selected top 3. For the selected features (*f*_*t*,*i*_, where *t* denotes set of features and *i* is index of feature in that set) we conducted contribution analysis. We calculated the contribution for each feature over the full range from its minimal to maximal value in *m* equally distributed points. The contribution analysis included the randomization process to create two instances as inputs to regression model. The first instance is a vector where each feature value is sampled at random from the data set *t*. The second instance differs in *i*^*th*^ feature which is not random but takes a particular value from set of previously defined *m* values that are currently under contribution analysis. The contribution of the feature is the difference between the outputs of the regression model produced using the first and the second instance as input. Due to the randomization process this procedure is repeated for a defined number of iterations. By averaging the results from all iterations, we obtained the final value for contribution. In addition to this value, we also report the standard deviation of the values obtained in each iteration, which provides information on the contribution stability and quantify complex interactions among features. We created plots (Fig. 5) for 12 features - top 3 for each of four data sets, ordered from left to right according to RFE ranks, sampled in *m* = 12 points with contributions calculated through 100 iterations. In addition, the 12 graphs that correspond to features ranked from 4^*th*^ to 6^*th*^ place for each data set are provided in the supplement - Fig. S2. Models where RFE selected less than 6 features (migration, activity) were just extend for the purpose of visualisation. All graphs contain points of the mean contribution and error bars in the length of standard deviation. Red color indicates points with feature values that are associated with increased HIV prevalence, and orange color indicates feature values that are associated with decreased HIV prevalence. The gray part of graph denotes the range where the standard distribution crosses zero, meaning that contribution is neither strongly positive nor negative.

Contributions of the three connectivity features are presented in Fig. 5(a–c). Top three features represent the communication flow expressed as the number of calls per resident of a department during the days of weekend in the time slots 01–02 AM, 02–03 AM and 03–04 AM, over a 5-month period. We can notice that the top connection features are related to weekend night-time communication and all have a positive slope. A similar graph (Fig. S2) is obtained for the 5^*th*^ ranked feature related to weekday 03–04 AM communication. According to the model, the departments with higher night-time communication have a higher prevalence rate. In further analysis of the contribution plot shown in Fig. 5(a), values higher than 0.2 can be seen as indicators of behavior increasing the risk of infection and thus critical for HIV. For example, for the department where this feature has the maximum value, the expectation of HIV prevalence is by 0.3 ± 0.15 higher than average. The plots for features ranked at 4^*th*^ and 6^*th*^ place (Fig. S2), refer to average call duration during the hours of early morning (06–07 AM) and contribute to HIV prevalence in a different way. The graphs with negative slope indicate that, for departments were people have longer talks early in the morning, we can expect lower HIV prevalence. We can observe this as a social signature^41^ and may hypothesize that longer talks early in the morning could be an indicator of emotionally close relationships and lower-risk behavior.

In the contribution analysis of spatial features, area and gyration stand out as features with higher impact. Area is measured over weekdays and gyration over weekday and weekend nights. The model suggests that departments where people tend to cover a larger area, have a higher HIV prevalence rate (Fig. 5(f)). This is also confirmed by the 4^*th*^ ranked feature, which measures the area covered over weekends (Fig. S2). Gyration, a measure of standard deviation from the mean location, negatively impacts HIV (Fig. 5(d,e) and also Fig. S2). But it is no surprise that small gyration indicates higher HIV, since it has already been shown in other studies that there is a higher expectation of shorter movements in the denser urban areas^42^, and those urban areas are usually more affected by HIV. When the area covered is tracked only during the hours of the night, the contribution graph has a negative slope as it does in the case of gyration (see graph for 5^*th*^ ranked feature - area covered during weekday nights, Fig. S2).

The contributions of overall in and out migration features are shown in Fig. 5(g,i). Both plots indicate that larger migration flows are associated to higher HIV prevalence. We can notice the strong impact of incoming migrations. For the department where this feature has the maximum value, the expectation of the HIV prevalence is by 1.0 ± 0.5 higher than the average. Among the top three features is the one that quantifies the number of outbound migrations per resident of a department, with the duration of staying for more than 10 days. Its contribution plot, presented in Fig. 5(h), shows negative impact. The plots for features ranked between 4^*th*^ and 6^*th*^ place (Fig. S2) further show that out migrations, with stays longer than one day have a positive slope, and those with stays longer than 5 or 9 days exhibit a negative slope. The contribution analysis of the migration features uncovers an interesting phenomenon. The overall amount of migrations is linked to higher HIV prevalence, and this positive slope remains true for migrations up to a few days, but beyond that, the slope becomes negative. The slope changes once the thresholds of 4 days for out migrations and 3 days for in migrations are reached. The model suggests that the risk comes from shorter stays at host departments and higher dynamics in migrations, while the longer stays are associated with lower HIV.

The contribution of the activity features, expressed through the number of calls and SMSs per residents of a department, are shown in Fig. 5(j–l). As with the connectivity features, night-time activity is strongly linked to HIV and higher activity implies higher prevalence rates. This is further confirmed by the 4^*th*^- and 5^*th*^-ranked features confirms that encompass activity during weekday nights, between 1 AM and 2 AM and weekend nights, between 4 AM and 5 AM. On the contrary, the feature ranked 6^*th*^, which refers to early morning activity (07–08 AM) has a negative slope.

RFE method helps us to identify the subset of stronger factors that have highest impact on HIV prevalence prediction. Contribution analysis further uncovers what the trained models learned from the data and allows us to compare them, analyze features and to make decisions concerning final model. RFE ranking differs from the naïve approach that orders features based on their individual relevance (see Methods, Recursive feature elimination subsection for further explanations). We can observe from Fig. 5 that features impacts measured through contribution are not ordered exactly as with RFE due to the interactions between features. Selected subset work in synergy to provide the prediction of the HIV prevalence. If we use only SVR-RFE model learned on spatial features, that means that we have to measure 6 selected features, make predictions and further estimate corresponding contributions. In case that we want to rely on combination of models, then we need coefficients used in stacking regressors. Estimated values for combining SVR-RFE models learned on connectivity, spatial, migration and activity features are 0.24, 0.27, 0.24 and 0.22 respectively. Features selection and contribution analysis could also serve for a new iteration of feature engineering. For example, top 3 activity features include night hours intervals 00–01, 01–02 and 02–03, and having similar contribution graphs they can be grouped into one that covers 00–03h interval. Evaluation reveals that grouping produced model with similar performance, but with lower complexity. In this way, we can search for better models. The resulting contribution plots can be also used to create new hypotheses in epidemiology, when disease distribution and spread are concerned, and, subsequently, to quantify the risk of increase in the prevalence of HIV.

## Discussion

The usage of the mobile phone data that can unveil patterns of human interactions and mobility, is gaining increased attention in epidemiology. In the study presented here, we placed the mobile phone data in the context of a generalized HIV epidemic. Raw data was processed searching for patterns that could explain the spatial variation in disease prevalence. We discovered that strong ties and hubs in the communication align with HIV hot spots. The strong ties created by user mobility revealed pathways that connect regions with higher prevalence. Abidjan, the city most severely affected by HIV - emerged as the center of migrations.

Next, we focused on extracting features related to the connectivity and mobility of users at the level of spatial units (departments) that could be used to predict HIV prevalence. Several regression methods were used to address that task, and the results obtained on a subset of departments, for which good estimates of HIV prevalence exist, are promising and can lead to generation of new hypotheses. The initial set of 224 features was reduced using a recursive feature elimination procedure, allowing us to identify features with the largest impact on prediction. It turned out that night-time connectivity and activity, area coverd by users and overall migrations are strongly linked to HIV prevalence. Models built on spatial features (gyration, area, perimeter of convex hull, diameter and distance) exhibit high predictive power (*ρ* = 0.753, *RRMSE* = 0.294). Future work should include a detailed analysis of spatio-temporal dynamics of human motion in the context of primary and subsidiary habitats^43^, where the first denote frequently visited locations during typical daily activities and the second capture additional travel.

A few real world implications emerged from the obtained results. The first is in the possibility to use machine learning algorithms coupled with feature engineering for predicting disease prevalence. HIV surveys are expensive and difficult to carry out. Rather than increasing the sampling size of a survey at the level of whole country, only representative parts could be sampled in such way to provide good estimates of HIV for selected spatial units. Then predictive model could be learned, evaluated and finally applied to the rest of the country to generalize on unobserved spatial units. This would enable faster access to HIV estimates. The second implication arises from recent study^44^ on geospatial modeling of rollout plans for antiretroviral drugs allocation. The study reveals that utilitarian plan in the allocation of constrained resources could prevent more infections than egalitarian plan, i.e. geographic targeting is better than geographic equity. Underlying optimization ranks spatial units based on the efficiency of interventions and HIV incidence rates. Features and predictions inferred from mobile phone data can be also included into strategy for geographic resource allocation and thus help with epidemic control. Integrating mobility and connectivity patterns into decisions making process for resources allocation would be beneficial especially in adjusting the plan over the time as new resources become available and change in HIV incidences, mobility and connectivity patterns occur. The third implication targets epidemiologist. The purpose of our study was to draw attention of HIV epidemiologist to rich source of information such as mobile phone data that are constantly collecting by service phone providers and nowadays becoming available for other African countries^45^. Our results shed light on HIV epidemiology in Ivory Coast, but our intent was not to provide a final model for HIV prediction, rather we put our efforts to explore numerous features and different models that would allow epidemiologist to gain insights, make new hypothesis and enrich their studies. Final model should be selected together with epidemiologist, by taking into account complexity, relevant features and background knowledge. Further evaluations could be extended on other African countries with generalized epidemiology.

The limitations of our study arise from spatial and temporal scale of data. On one side, HIV data is limited by the measurement strategy of DHS, UNAIDS or other relevant entities. The quality and spatial resolution of such data are determined by the sampling design - frequency and distribution of measurements. The variability in HIV prevalence across the Ivory Coast is certainly higher than one modelled on the department level, but we lacked more precise measurements to account for it better. The time resolution is even scarcer. HIV measurement campaigns are organized only once every few years (for the Ivory Coast 2012, 2005, 2001). Our findings linked aggregated behavioral patterns to HIV prevalence rates, but discovered correlations do not imply causation. To explore causation, we would need more estimates on changes of HIV prevalence during time. This could soon be overcome by a new device that easily connects to a smartphone^46^. The device performs the ELISA test and discovers disease markers from a tiny drop of blood, taken from a finger, in just 15 minutes. This approach has a high acceptance rate among population and will enable large–scale screening.

Another important source of information for studying HIV epidemiology is the demography of the population. While data on the population size are available in the high resolution, when it comes to the structure (age, gender…) resolution is coarse. Age and gender distribution across 50 departments of the Ivory Coast is missing. Corresponding HIV prevalences were estimated under assumption that departments’ demography (age) was uniform across country and identical to the age distribution of the overall population^47^. It is possible that features used in regression models reflect hidden demographic variables, e.g., areas with nighttime high number of calls could be those with a younger demographic. But adjusting our regression models for the demography is currently not feasible. One possibility for further research is to rely on machine learning algorithms to infer demographic attributes of users^48^,^49^. For that we would need consent from smaller sample of users to use their attributes for learning the model.

On the other hand, the spatial resolution of mobile phone data is restricted by the distribution of carrier’s antennas and the time resolution is conditioned by users’ phone activity (calls or massages). But the major constraint on using mobile phone data are the privacy concerns^50^. Beside the mandatory user anonymization, mobile phone data are, usually, further spatially and/or temporally aggregated, or a part of information is removed. For example, the antennas are aggregated at the level of larger geographical units, time is expressed in hourly intervals, and communication graphs at the level of users are detached from any spatial information. In D4D data sources, mobile phone data sets are temporally aggregated to one-hour time slots, with preserved spatial resolution of 1250 antennas or spatially restricted to 255 sub-prefectures, but without time aggregation. Even with data aggregation, mobile phone data is still quite a richer source of information when compared to HIV estimates that are available across 50 departments. Only in the case of individual communication graphs (D4D SET4) where spatial information is completely removed, we lose any chance to link it with the HIV distribution. Those communication graphs, if geographically determined, would be an immense source of information for uncovering the connectivity at a more detailed scale. If such data becomes available in a privacy-acceptable form, further progress in the domain of modelling the spread of communicable diseases^51^ will be enabled.

In summary, our study showed how raw real world data can be used for significant knowledge extraction. We believe that our work, a first attempt to link mobile phone data and HIV epidemiology, lays a foundation for further research into ways to explain the heterogeneity of HIV and build predictive tools aimed at advancing public–health campaigns and decision making for HIV interventions. Together with other “big data” approaches to HIV epidemiology^52^ that rely on Twitter data^53^ and social networks^54^,^55^ our work fits well into the wider initiative of digital epidemiology^20^.

## Methods

### Data sources

#### Population data

We used the data set available from AfriPop project^29^, which contains full details on population distribution, summarized on the country level. The authors developed a new high resolution population distribution data set for Africa and analyzed rural accessibility to population centers. Contemporary population data was combined with detailed satellite-derived settlement in order to link the population distribution with the finest spatial resolution.

#### HIV data

Demographic and Health Surveys (DHS) provides data about the health status of countries. We used data collected in the survey conducted during 2011 and 2012^3^. This data provides estimates for ten administrative regions of the Ivory Coast. The results of the estimation are shown in Fig. 1(a).

#### D4D data

Mobile phone data sets originate from the Orange service provider in the Ivory Coast collected in the period from December 5 to April 22, 2012. and are further processed into four different D4D sets^23^. SET1 contains the antenna-to-antenna communication traffic flow of five million Orange costumers aggregated to hourly intervals. Each record contains the originating and terminating antennas of calls, the number of calls and overall duration. SET2 observes users in consecutive two-week periods, which do not significantly influence HIV transmission patterns. On the other hand, insight into the long term mobility (5 months long observation period) is possible trough SET3. Spatial resolution in this set is reduced from towers to sub-prefectures (255 spatial units). A record in this set contains the user id, time stamp and sub-prefecture ids. Although SET4 provides connectivity at the level of single users and could be very informative for HIV epidemiology, it lacks spatial information. We were not able to approximate users’ home location because IDs could not be related to the IDs in the second or third set.

Two of these were used in our study: SET1 and SET3 over entire available period. Although four days (5th December and 10th, 15th and 19th April), have about an order of magnitude less number of records than neighboring days, we evaluated that missing data negligibly impact extracted features and predictive models. Our features are defined either as sums of CDRs normalized by the population sizes or robust statistics (95 percentile), both derived over the full time period. Communication records for four above mentioned days account for less than 1% of overall number of records, varying from 0.05 to 0.73% across departments. For example value of feature related to overall communication in department with ID 1 (Fig. S1) is 164.4 compared to 164.3 obtained when days with missing data are removed. In case that CDR data have higher percentage of missing data (>5%) we recommend removing those days before calculating features.

Another issue with the data arises from sub-prefectures with no cell phone towers. But in our study, to align with HIV data, spatial resolution of both mobile phone data sets was reduced at the level of 50 departments by assigning towers (SET1) and sub-perfectures (SET3) to the departments to which they belong. Number of towers varies from 2 for northernmost department (ID 44, Fig. S1) to 454 for department 2 that encompasses largest city Abidjan. If HIV data with higher resolution become available, populations from sub-perfectures without towers should be redistributed across neighbouring sub-perfectures.

### Estimates on HIV prevalence at the level of departments

National estimates on HIV prevalence hide the heterogeneity that exists within the country. To unveil subnational prevalence rates, a recently proposed method - prevR^56^ relies on an estimation function and DHS measurements to generate a surface of HIV prevalence. Estimations are based on Gaussian kernel density functions with adaptive bandwidths. An estimate on HIV prevalence in a spatial point (*x*, *y*) is determined by Eq. 1.


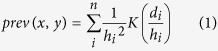


where, n is the number of samples, *d*_*i*_ the geometrical distance between sample *i* and point (*x*,  *y*), *K* is the kernel function and *h*_*i*_ the bandwidth used for sample *i*. Additionally, an indicator of the quality of the estimates was assigned to each department, based on the survey sampling size^47^. Some estimates are very uncertain and should be interpreted with caution. See supplement Table S1 for estimated values and quality indicators.

### Strong ties identification

Estimating social tie strengths among people and classifying them as strong or weak helps in understanding socio-geographical relations^57^. In our study, we aim at quantifying tie strengths among departments and identifying the strong ones. Ties between departments *i* and *j* are expressed by communication or mobility flow *ω*_*ij*_ directed from *i* to *j* and quantified as number of calls or mobilities originating from *i* and terminating at *j* divided by the population of originating department. To categorize those connectivity ties as strong or weak we adopted the approach from^58^ where Eq. 2 is used as disparity filter for detecting the significant links.


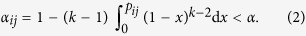


where *i* and *j* are indexes of department, *α*_*ij*_ is the significance of tie from department *i* to department *j*, *k* is the degree of a node under consideration and *p*_*ij*_ = *ω*_*ij*_/*s*_*i*_ corresponds to weights normalized by the node (department) strength 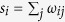. Degree *k* of each node in our communication and mobility graphs is 49, inner loops are not taken into account. Ties with *α*_*ij*_ < *α* are classified as strong ties, statistically significant at the level *α*. Underlying null hypothesis used in significance inference assumes that normalized weights of edges linking node *i* with its neighbours are produced by random assignment from uniform distribution. In our experiment statistical significance *α* is set at 0.01. The filter works locally at the level of nodes that globally allows to preserve relevant fluctuations at different scales. We applied version of the disparity filter for directed weighted networks that considers incoming and outgoing links associated to a node separately. After filtering procedure, for the purpose of visualization, directed graph was simply transformed into undirected by summing *ω*_*ij*_ and *ω*_*ji*_.

### Ridge regression

Ridge regression is a variant of ordinary multiple linear regression whose goal is to circumvent the problem of instability, arising, among other, from co-linearity of the predictor variables. It works with the original variables and tries to minimize penalized sum of squares. Like the ordinary least squares, ridge regression includes all predictor variables, but typically with smaller coefficients, depending upon the value of the complexity parameter *λ*. The selection of the ridge parameter *λ* plays an important role, it multiplies the ridge penalty and thus controls the strength of the shrinkage of coefficients toward zero^34^. The value of *λ* is estimated though leave-one-out validation. Estimated *λ* for the best models learned on subset of departments with good and moderate HIV estimates are 0.1, 0.1, 0.05, 0.02 for connectivity, spatial, migration and activity features, respectively. For models learned on full set of departments estimated values are 0.05, 0.05, 0.1 and 0.01.

### Support vector regression

Support vector machines are a set of supervised learning methods used for classification and regression analysis. A version of SVM for regression analysis is the Support Vector Regression (SVR)^35^. SVR searches for the optimal regression function, but allows a tolerance margin (*ε*), creating a tube around the regression function where errors in predictions on training data are ignored. The method also includes a regularization parameter in the form of a cost parameter (*C*), that penalizes the training errors outside the tube. In our experiments we used a linear kernel, the default *ε* = 0.1, while the value of *C* was estimated though leave-one-out validation. Estimated *C* for the best models learned on subset of departments with good and moderate HIV estimates are 0.2, 0.5, 1.0 and 0.1 for connectivity, spatial, migration and activity features, respectively. For models learned on full set of departments estimated values are 0.06, 3.0, 4.0 and 1.0.

### Recursive Feature Elimination

Recursive Feature Elimination (RFE) is a greedy method for selecting a defined number of features. It starts from the initial set of features and builds a model (in our case SVM or Ridge), assigns weights to each feature based on estimate from the predictive model, eliminates the lowest ranked feature and then recursively repeats this procedure on the remaining set of features until it reaches the desired number of features. The output is a top-ranked feature subset obtained through this recursive procedure^36^.

In the context of linear SVR and Ridge regression, weights of the features in each step of RFE iteration correspond to the coefficients assigned by SVR or Ridge model on candidate feature subset that is under evaluation in recursive process. RFE differs from the naïve ranking. While naïve orders features based on their individual relevance, RFE searches for feature subset that contain complementary, but not necessarily individually most relevant features. In this way, RFE can identify important interactions between features and outperform selection according to naïve ranking. This is particularly evident in the case of correlated features.

## Additional Information

**How to cite this article**: Brdar, S. *et al.* Unveiling Spatial Epidemiology of HIV with Mobile Phone Data. *Sci. Rep.* **6**, 19342; doi: 10.1038/srep19342 (2016).

## Supplementary Material

Supplementary Information

## Figures and Tables

**Table 1:** Evaluation of predictive models on good and moderate HIV estimates - Correlation coefficient (Relative Root Mean Square Error): *ρ* (*RRMSE*).

Features	Predictive models			
	Ridge	Ridge + RFE	SVR	SVR + RFE
Connectivity features (SET1)	0.624 (0.331)	0.626 (0.331)	0.661 (0.306)	**0.669 (0.301)**
Spatial features (SET3)	0.639 (0.434)	0.703 (0.376)	0.544 (0.351)	**0.753 (0.294)**
Migration features (SET3)	0.585 (0.369)	0.585 (0.369)	0.678 (0.307)	**0.691 (0.288)**
Activity features (SET3)	0.618 (0.339)	0.645 (0.325)	0.633 (0.316)	**0.664 (0.302)**
Ensemble	0.610 (0.327)	0.601 (0.327)	0.659 (0.305)	**0.710 (0.287)**
Best Random	−0.231 (0.511)	−0.066 (0.480)	−0.065 (0.479)	0.070 (0.441)

**Table 2:** Evaluation of predictive models on all HIV estimates - Correlation coefficient (Relative Root Mean Square Error): *ρ* (*RRMSE*).

Features	Predictive models			
	Ridge	Ridge + RFE	SVR	SVR + RFE
Connectivity features (SET1)	0.467 (0.556)	0.481 (0.546)	0.501 (0.516)	**0.508 (0.514)**
Spatial features (SET3)	0.363 (0.540)	**0.431 (0.523)**	**0.310 (0.552)**	**0.336 (0.545)**
Migration features (SET3)	0.269 (0.630)	0.315 (0.613)	0.291 (0.637)	**0.375 (0.599)**
Activity features (SET3)	0.511 (0.542)	0.542 (0.535)	0.522 (0.537)	**0.627 (0.509)**
Ensemble	0.500 (0.527)	**0.543** (0.519)	0.535 (0.515)	0.518 **(0.514)**
Best Random	0.020 (0.760)	0.202 (0.657)	0.139 (0.630)	0.038 (0.607)
