# Supplementary material for: Unveiling Spatial Epidemiology of HIV with Mobile Phone Data
Source: Sci Rep. 2016 Jan 13;6:19342. doi: 10.1038/srep19342 (PMC4725841; doi:10.1038/srep19342)
Supplement: Supplementary Information [file srep19342-s1.pdf]

Unveiling Spatial Epidemiology of HIV with Mobile Phone Data
Supplementary Information

Sanja Brdar[1,+], Katarina Gavrić [1,+], Dubravko Ćulibrk[1,2], and Vladimir Crnojević [1]

[1]Faculty of Technical Sciences, University of Novi Sad, Serbia
[2]Department of Information Engineering and Computer Science, University of Trento, Italy
[+]these authors contributed equally to this work

August 11, 2015

In this document we provide following supplementary information:

Ivory Coast departments map

Figure S1 shows boundaries of Ivory Coast departments with corresponding *id* numbers used in our study.

Features Description

Tables S2 and S3 display the feature names and their descriptions. The marked features (*) are those included in the best SVR-RFE models.

HIV estimates

HIV estimates at department level from national population-based surveys (source Joseph Larmarange and Victoria Bendaud, AIDS 2014) are provided in Table S1.

Features Contribution

Figure S2 reveals results of features contribution analysis for features ranked from 4th to 6th place in recursive feature elimination procedure.

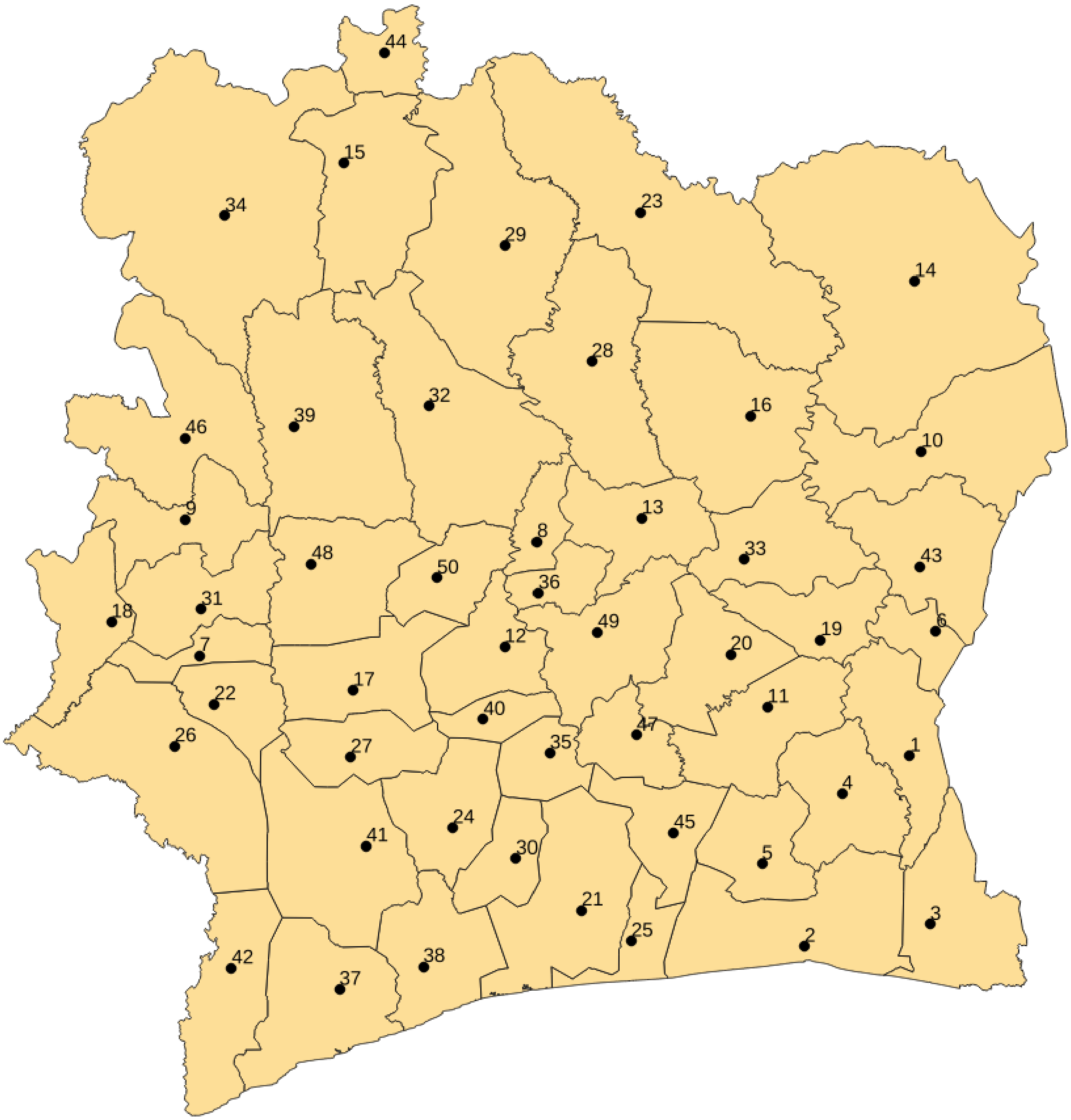

Figure S1: Departments of Ivory Coast. We used open source QGIS software (http://www.qgis.org) to create map.

Table S1: HIV prevalence estimates with quality indicators

Department ID	Department Name	HIV prevalence	Quality
1	Abengourou	2.70	good
2	Abidjan	3.60	good
3	Aboisso	3.20	uncertain
4	Adzopé	4.70	uncertain
5	Agboville	3.10	uncertain
6	Agnibilkrou	3.20	moderately good
7	Bangolo	3.30	uncertain
8	Béoumi	2.50	uncertain
9	Biankouma	2.00	uncertain
10	Bondoukou	1.70	moderately good
11	Bongouanou	3.30	uncertain
12	Bouaflé	2.50	uncertain
13	Bouaké	3.10	good
14	Bouna	2.00	uncertain
15	Boundiali	1.40	uncertain
16	Dabakala	2.50	uncertain
17	Daloa	1.90	uncertain
18	Danané	2.10	uncertain
19	Daoukro	3.80	uncertain
20	Dimbokro	2.30	uncertain
21	Divo	2.00	uncertain
22	Duékoué	3.80	uncertain
23	Ferkessédougou	2.90	uncertain
24	Gagnoa	2.00	uncertain
25	Grand-Lahou	2.20	uncertain
26	Guiglo	3.20	moderately good
27	Issia	1.90	uncertain
28	Katiola	4.00	uncertain
29	Korhogo	2.30	good
30	Lakota	2.00	uncertain
31	Man	2.80	moderately good
32	Mankono	2.10	moderately good
33	Mbahiakro	3.00	uncertain
34	Odienné	1.70	moderately good
35	Oumé	2.50	uncertain
36	Sakassou	1.70	uncertain
37	San-Pédro	3.30	moderately good
38	Sassandra	3.50	uncertain
39	Séguéla	1.80	uncertain
40	Sinfra	2.50	uncertain
41	Soubré	2.80	moderately good
42	Tabou	5.70	uncertain
43	Tanda	2.10	moderately good
44	Tengréla	0.60	uncertain
45	Tiassalé	2.60	uncertain
46	Touba	1.60	moderately good
47	Toumodi	2.60	uncertain
48	Vavoua	1.30	uncertain
49	Yamoussoukro	3.10	moderately good
50	Zuénoula	1.10	uncertain

Table S2: Features descriptions for SET1.

Feature Name	Description
Connectivity-Inner*	Sum of inner calls in department divided by population of department
Connectivity-Orig*	Sum of calls originating from department divided by population of department
Connectivity-Term*	Sum of calls terminating in department divided by population of department
Connectivity-All*	Sum of all calls in department divided by population of department
Connectivity-Weekday*	Sum of all calls in department during weekdays divided by population of department
Connectivity-Weekend	Sum of all calls in department during weekends divided by population of department
Connectivity-Weekday-00-08h	Sum of all calls in department during weekdays in 00-08h time interval divided by population of department
Connectivity-Weekday-08-16h*	Sum of all calls in department during weekdays in 08-16h time interval divided by population of department
Connectivity-Weekday-16-24h*	Sum of all calls in department during weekdays in 16-24h time interval divided by population of department
Connectivity-Weekend-00-08h*	Sum of all calls in department during weekends in 00-08h time interval divided by population of department
Connectivity-Weekend-08-16h	Sum of all calls in department during weekends in 08-16h time interval divided by population of department
Connectivity-Weekend-16-24h	Sum of all calls in department during weekends in 16-24h time interval divided by population of department
Connectivity-Weekday-00h	Sum of all calls in department during weekdays in 00-01h time interval divided by population of department
Connectivity-Weekday-01h*	Sum of all calls in department during weekdays in 01-02h time interval divided by population of department
Connectivity-Weekday-02h*	Sum of all calls in department during weekdays in 02-03h time interval divided by population of department
Connectivity-Weekday-03h*	Sum of all calls in department during weekdays in 03-04h time interval divided by population of department
Connectivity-Weekday-04h	Sum of all calls in department during weekdays in 04-05h time interval divided by population of department
Connectivity-Weekday-05h	Sum of all calls in department during weekdays in 05-06h time interval divided by population of department
Connectivity-Weekday-06h*	Sum of all calls in department during weekdays in 06-07h time interval divided by population of department
Connectivity-Weekday-07h	Sum of all calls in department during weekdays in 07-08h time interval divided by population of department
Connectivity-Weekday-08h*	Sum of all calls in department during weekdays in 08-09h time interval divided by population of department
Connectivity-Weekday-09h*	Sum of all calls in department during weekdays in 09-10h time interval divided by population of department
Connectivity-Weekday-10h*	Sum of all calls in department during weekdays in 10-11h time interval divided by population of department
Connectivity-Weekday-11h*	Sum of all calls in department during weekdays in 11-12h time interval divided by population of department
Connectivity-Weekday-12h*	Sum of all calls in department during weekdays in 12-13h time interval divided by population of department

Feature Name	Description
Connectivity-Weekday-13h*	Sum of all calls in department during weekdays in 13-14h time interval divided by population of department
Connectivity-Weekday-14h*	Sum of all calls in department during weekdays in 14-15h time interval divided by population of department
Connectivity-Weekday-15h*	Sum of all calls in department during weekdays in 15-16h time interval divided by population of department
Connectivity-Weekday-16h*	Sum of all calls in department during weekdays in 16-17h time interval divided by population of department
Connectivity-Weekday-17h*	Sum of all calls in department during weekdays in 17-18h time interval divided by population of department
Connectivity-Weekday-18h	Sum of all calls in department during weekdays in 18-19h time interval divided by population of department
Connectivity-Weekday-19h	Sum of all calls in department during weekdays in 19-20h time interval divided by population of department
Connectivity-Weekday-20h	Sum of all calls in department during weekdays in 20-21h time interval divided by population of department
Connectivity-Weekday-21h	Sum of all calls in department during weekdays in 21-22h time interval divided by population of department
Connectivity-Weekday-22h	Sum of all calls in department during weekdays in 22-23h time interval divided by population of department
Connectivity-Weekday-23h*	Sum of all calls in department during weekdays in 23-00h time interval divided by population of department
Connectivity-Weekend-00h*	Sum of all calls in department during weekends in 00-01h time interval divided by population of department
Connectivity-Weekend-01h*	Sum of all calls in department during weekends in 01-02h time interval divided by population of department
Connectivity-Weekend-02h*	Sum of all calls in department during weekends in 02-03h time interval divided by population of department
Connectivity-Weekend-03h*	Sum of all calls in department during weekends in 03-04h time interval divided by population of department
Connectivity-Weekend-04h	Sum of all calls in department during weekends in 04-05h time interval divided by population of department
Connectivity-Weekend-05h	Sum of all calls in department during weekends in 05-06h time interval divided by population of department
Connectivity-Weekend-06h*	Sum of all calls in department during weekends in 06-07h time interval divided by population of department
Connectivity-Weekend-07h*	Sum of all calls in department during weekends in 07-08h time interval divided by population of department
Connectivity-Weekend-08h	Sum of all calls in department during weekends in 08-09h time interval divided by population of department
Connectivity-Weekend-09h*	Sum of all calls in department during weekends in 09-10h time interval divided by population of department
Connectivity-Weekend-10h*	Sum of all calls in department during weekends in 10-11h time interval divided by population of department
Connectivity-Weekend-11h*	Sum of all calls in department during weekends in 11-12h time interval divided by population of department
Connectivity-Weekend-12h	Sum of all calls in department during weekends in 12-13h time interval divided by population of department
Connectivity-Weekend-13h	Sum of all calls in department during weekends in 13-14h time interval divided by population of department
Connectivity-Weekend-14h	Sum of all calls in department during weekends in 14-15h time interval divided by population of department

Feature Name	Description
Connectivity-Weekend-15h	Sum of all calls in department during weekends in 15-16h time interval divided by population of department
Connectivity-Weekend-16h	Sum of all calls in department during weekends in 16-17h time interval divided by population of department
Connectivity-Weekend-17h*	Sum of all calls in department during weekends in 17-18h time interval divided by population of department
Connectivity-Weekend-18h	Sum of all calls in department during weekends in 18-19h time interval divided by population of department
Connectivity-Weekend-19h	Sum of all calls in department during weekends in 19-20h time interval divided by population of department
Connectivity-Weekend-20h	Sum of all calls in department during weekends in 20-21h time interval divided by population of department
Connectivity-Weekend-21h	Sum of all calls in department during weekends in 21-22h time interval divided by population of department
Connectivity-Weekend-22h	Sum of all calls in department during weekends in 22-23h time interval divided by population of department
Connectivity-Weekend-23h	Sum of all calls in department during weekends in 23-00h time interval divided by population of department
Average-Inner-Call-Duration	Sum of durations of inner calls in department divided by number of calls
Average-Orig-Call-Duration	Sum of durations of originating calls in department divided by number of calls
Average-Term-Call-Duration	Sum of durations of originating calls in department divided by number of calls
Average-Call-Duration	Sum of durations of all calls in department divided by number of calls
Average-Call-Duration-Weekday	Sum of durations of all calls in department during weekdays divided by number of calls
Average-Call-Duration-Weekend*	Sum of durations of all calls in department during weekends divided by number of calls
Average-Call-Duration-Weekday-00-08h*	Sum of durations of all calls in department during weekdays in time interval 00-08h divided by number of calls
Average-Call-Duration-Weekday-08-16h	Sum of durations of all calls in department during weekdays in time interval 08-16h divided by number of calls
Average-Call-Duration-Weekday-16-24h	Sum of durations of all calls in department during weekdays in time interval 16-24h divided by number of calls
Average-Call-Duration-Weekend-00-08h*	Sum of durations of all calls in department during weekends in time interval 00-08h divided by number of calls
Average-Call-Duration-Weekend-08-016h*	Sum of durations of all calls in department during weekends in time interval 08-16h divided by number of calls
Average-Call-Duration-Weekend-16-24h	Sum of durations of all calls in department during weekends in time interval 16-24h divided by number of calls
Average-Call-Duration-Weekday-00h	Sum of durations of all calls in department during weekdays in time interval 00-01h divided by number of calls
Average-Call-Duration-Weekday-01h*	Sum of durations of all calls in department during weekdays in time interval 01-02h divided by number of calls
Average-Call-Duration-Weekday-02h*	Sum of durations of all calls in department during weekdays in time interval 02-03h divided by number of calls
Average-Call-Duration-Weekday-03h*	Sum of durations of all calls in department during weekdays in time interval 03-04h divided by number of calls
Average-Call-Duration-Weekday-04h	Sum of durations of all calls in department during weekdays in time interval 04-05h divided by number of calls

Feature Name	Description
Average-Call-Duration-Weekday-05h*	Sum of durations of all calls in department during weekdays in time interval 05-06h divided by number of calls
Average-Call-Duration-Weekday-06h*	Sum of durations of all calls in department during weekdays in time interval 06-07h divided by number of calls
Average-Call-Duration-Weekday-07h*	Sum of durations of all calls in department during weekdays in time interval 07-08h divided by number of calls
Average-Call-Duration-Weekday-08h	Sum of durations of all calls in department during weekdays in time interval 08-09h divided by number of calls
Average-Call-Duration-Weekday-09h	Sum of durations of all calls in department during weekdays in time interval 09-10h divided by number of calls
Average-Call-Duration-Weekday-10h	Sum of durations of all calls in department during weekdays in time interval 10-11h divided by number of calls
Average-Call-Duration-Weekday-11h	Sum of durations of all calls in department during weekdays in time interval 11-12h divided by number of calls
Average-Call-Duration-Weekday-12h*	Sum of durations of all calls in department during weekdays in time interval 12-13h divided by number of calls
Average-Call-Duration-Weekday-13h*	Sum of durations of all calls in department during weekdays in time interval 13-14h divided by number of calls
Average-Call-Duration-Weekday-14h	Sum of durations of all calls in department during weekdays in time interval 14-15h divided by number of calls
Average-Call-Duration-Weekday-15h	Sum of durations of all calls in department during weekdays in time interval 15-16h divided by number of calls
Average-Call-Duration-Weekday-16h	Sum of durations of all calls in department during weekdays in time interval 16-17h divided by number of calls
Average-Call-Duration-Weekday-17h	Sum of durations of all calls in department during weekdays in time interval 17-18h divided by number of calls
Average-Call-Duration-Weekday-18h	Sum of durations of all calls in department during weekdays in time interval 18-19h divided by number of calls
Average-Call-Duration-Weekday-19h	Sum of durations of all calls in department during weekdays in time interval 19-20h divided by number of calls
Average-Call-Duration-Weekday-20h	Sum of durations of all calls in department during weekdays in time interval 20-21h divided by number of calls
Average-Call-Duration-Weekday-21h*	Sum of durations of all calls in department during weekdays in time interval 21-22h divided by number of calls
Average-Call-Duration-Weekday-22h	Sum of durations of all calls in department during weekdays in time interval 22-23h divided by number of calls
Average-Call-Duration-Weekday-23h*	Sum of durations of all calls in department during weekdays in time interval 23-00h divided by number of calls
Average-Call-Duration-Weekend-00h	Sum of durations of all calls in department during weekends in time interval 00-01h divided by number of calls
Average-Call-Duration-Weekend-01h*	Sum of durations of all calls in department during weekends in time interval 01-02h divided by number of calls
Average-Call-Duration-Weekend-02h*	Sum of durations of all calls in department during weekends in time interval 02-03h divided by number of calls
Average-Call-Duration-Weekend-03h*	Sum of durations of all calls in department during weekends in time interval 03-04h divided by number of calls
Average-Call-Duration-Weekend-04h	Sum of durations of all calls in department during weekends in time interval 04-05h divided by number of calls
Average-Call-Duration-Weekend-05h*	Sum of durations of all calls in department during weekends in time interval 05-06h divided by number of calls
Average-Call-Duration-Weekend-06h*	Sum of durations of all calls in department during weekends in time interval 06-07h divided by number of calls

Feature Name	Description
Average-Call-Duration-Weekend-07h*	Sum of durations of all calls in department during weekends in time interval 07-08h divided by number of calls
Average-Call-Duration-Weekend-08h*	Sum of durations of all calls in department during weekends in time interval 08-09h divided by number of calls
Average-Call-Duration-Weekend-09h	Sum of durations of all calls in department during weekends in time interval 09-10h divided by number of calls
Average-Call-Duration-Weekend-10h	Sum of durations of all calls in department during weekends in time interval 10-11h divided by number of calls
Average-Call-Duration-Weekend-11h	Sum of durations of all calls in department during weekends in time interval 11-12h divided by number of calls
Average-Call-Duration-Weekend-12h*	Sum of durations of all calls in department during weekends in time interval 12-13h divided by number of calls
Average-Call-Duration-Weekend-13h*	Sum of durations of all calls in department during weekends in time interval 13-14h divided by number of calls
Average-Call-Duration-Weekend-14h*	Sum of durations of all calls in department during weekends in time interval 14-15h divided by number of calls
Average-Call-Duration-Weekend-15h*	Sum of durations of all calls in department during weekends in time interval 15-16h divided by number of calls
Average-Call-Duration-Weekend-16h*	Sum of durations of all calls in department during weekends in time interval 16-17h divided by number of calls
Average-Call-Duration-Weekend-17h	Sum of durations of all calls in department during weekends in time interval 17-18h divided by number of calls
Average-Call-Duration-Weekend-18h	Sum of durations of all calls in department during weekends in time interval 18-19h divided by number of calls
Average-Call-Duration-Weekend-19h	Sum of durations of all calls in department during weekends in time interval 19-20h divided by number of calls
Average-Call-Duration-Weekend-20h	Sum of durations of all calls in department during weekends in time interval 20-21h divided by number of calls
Average-Call-Duration-Weekend-21h	Sum of durations of all calls in department during weekends in time interval 21-22h divided by number of calls
Average-Call-Duration-Weekend-22h	Sum of durations of all calls in department during weekends in time interval 22-23h divided by number of calls
Average-Call-Duration-Weekend-23h*	Sum of durations of all calls in department during weekends in time interval 23-00h divided by number of calls

Table S3: Features descriptions for SET3.

Feature Name	Description
Gyration-All	95 percentile of the distribution of user's radius of gyration for all visited locations
Gyration-Night*	95 percentile of the distribution of user's radius of gyration for locations visited during nigth hours 22h-05h
Gyration-Weekday	95 percentile of the distribution of user's radius of gyration for locations visited during weekdays
Gyration-Weekday-Night*	95 percentile of the distribution of user's radius of gyration for locations visited during weekdays nigth hours 22h-05h
Gyration-Weekend	95 percentile of the distribution of user's radius of gyration for locations visited during weekend hours
Gyration-Weekend-Night*	95 percentile of the distribution of user's radius of gyration for locations visited during weekend nigth hours 22h-05h
Diameter-All	95 percentile of the distribution of user's diameter of convex hull for all visited locations

Feature Name	Description
Diameter-Night	95 percentile of the distribution of user's diameter of convex hull for locations visited during nigth hours 22h-05h
Diameter-Weekday	95 percentile of the distribution of user's diameter of convex hull for locations visited during weekdays
Diameter-Weekday-Night	95 percentile of the distribution of user's diameter of convex hull for locations visited during weekdays nigth hours 22h-05h
Diameter-Weekend	95 percentile of the distribution of user's diameter of convex hull for locations visited during weekend hours
Diameter-Weekend-Night	95 percentile of the distribution of user's diameter of convex hull for locations visited during weekend night hours 22h-05h
Area-All	95 percentile of the distribution of user's area of convex hull for all visited locations
Area-Night	95 percentile of the distribution of user's area of convex hull for locations visited during night hours 22h-05h
Area-Weekday*	Locations visited during weekday hours
Area-Weekday-Night*	Locations visited during weekday night hours
Area-Weekend*	Locations visited during weekend hours
Area-Weekend-Night	Locations visited during weekend night hours
Perimeter-All	95 percentile of the distribution of user's perimeter of convex hull for all visited locations
Perimeter-Night	95 percentile of the distribution of user's perimeter of convex hull during night hours
Perimeter-Weekday	95 percentile of the distribution of user's perimeter of convex hull during weekday hours
Perimeter-Weekday-Night	95 percentile of the distribution of user's perimeter of convex hull during weekday night hours
Perimeter-Weekend	95 percentile of the distribution of user's perimeter of convex hull during weekend hours
Perimeter-Weekend-Night	95 percentile of the distribution of user's perimeter of convex hull during weekend night hours
Distance-All	95 percentile of the distribution of user's sum of distances derived from sequences of visited locations
Out-Migration-Overall*	Sum of all users' mobilities out of home department divided by population of home department
Out-Migration-1day	Sum of users' mobilities out of home department that last more than 1 day divided by population of home department
Out-Migration-2days	Sum of users' mobilities out of home department that last more than 2 days divided by population of home department
Out-Migration-3days	Sum of users' mobilities out of home department that last more than 3 days divided by population of home department
Out-Migration-4days	Sum of users' mobilities out of home department that last more than 4 days divided by population of home department
Out-Migration-5days	Sum of users' mobilities out of home department that last more than 5 days divided by population of home department
Out-Migration-6days	Sum of users' mobilities out of home department that last more than 6 days divided by population of home department
Out-Migration-7days	Sum of users' mobilities out of home department that last more than 7 days divided by population of home department
Out-Migration-8days	Sum of users' mobilities out of home department that last more than 8 days divided by population of home department
Out-Migration-9days	Sum of users' mobilities out of home department that last more than 9 days divided by population of home department

Feature Name	Description
Out-Migration-10days*	Sum of users' mobilities out of home department that last more than 10 days divided by population of home department
In-Migration-Overall*	Sum of all users' mobilities into observed department from other departments divided by corresponding populations
In-Migration-1day	Sum of all users' mobilities into observed department from other departments that last more than 1 day divided by corresponding populations
In-Migration-2day	Sum of all users' mobilities into observed department from other departments that last more than 2 day divided by corresponding populations
In-Migration-3day	Sum of all users' mobilities into observed department from other departments that last more than 3 day divided by corresponding populations
In-Migration-4day	Sum of all users' mobilities into observed department from other departments that last more than 4 day divided by corresponding populations
In-Migration-5day	Sum of all users' mobilities into observed department from other departments that last more than 5 day divided by corresponding populations
In-Migration-6day	Sum of all users' mobilities into observed department from other departments that last more than 6 day divided by corresponding populations
In-Migration-7day	Sum of all users' mobilities into observed department from other departments that last more than 7 day divided by corresponding populations
In-Migration-8day	Sum of all users' mobilities into observed department from other departments that last more than 8 day divided by corresponding populations
In-Migration-9days	Sum of all users' mobilities into observed department from other departments that last more than 9 day divided by corresponding populations
In-Migration-10days	Sum of all users' mobilities into observed department from other departments that last more than 10 days divided by corresponding populations
Activity-All	Sum of all users' activities (calls/SMS) in department divided by population of department
Activity-Weekday	Sum of all users' activities (calls/SMS) in department during weekdays divided by population of department
Activity-Weekend	Sum of all users' activities (calls/SMS) in department during weekends divided by population of department
Activity-Weekday-00-08h	Sum of all users' activities (calls/SMS) in department during weekdays in 00-08h time interval divided by population of department
Activity-Weekday-08-16h	Sum of all users' activities (calls/SMS) in department during weekdays in 08-16h time interval divided by population of department
Activity-Weekday-16-24h	Sum of all users' activities (calls/SMS) in department during weekdays in 16-24h time interval divided by population of department
Activity-Weekend-00-08h	Sum of all users' activities (calls/SMS) in department during weekends in 00-08h time interval divided by population of department

Feature Name	Description
Activity-Weekend-08-016h	Sum of all users' activities (calls/SMS) in department during weekends in 08-16h time interval divided by population of department
Activity-Weekend-16-24h	Sum of all users' activities (calls/SMS) in department during weekends in 16-24h time interval divided by population of department
Activity-Weekday-00h*	Sum of all users' activities (calls/SMS) in department during weekdays in 00-01h time interval divided by population of department
Activity-Weekday-01h	Sum of all users' activities (calls/SMS) in department during weekdays in 01-02h time interval divided by population of department
Activity-Weekday-02h	Sum of all users' activities (calls/SMS) in department during weekdays in 02-03h time interval divided by population of department
Activity-Weekday-03h	Sum of all users' activities (calls/SMS) in department during weekdays in 03-04h time interval divided by population of department
Activity-Weekday-04h	Sum of all users' activities (calls/SMS) in department during weekdays in 04-05h time interval divided by population of department
Activity-Weekday-05h	Sum of all users' activities (calls/SMS) in department during weekdays in 05-06h time interval divided by population of department
Activity-Weekday-06h	Sum of all users' activities (calls/SMS) in department during weekdays in 06-07h time interval divided by population of department
Activity-Weekday-07h	Sum of all users' activities (calls/SMS) in department during weekdays in 07-08h time interval divided by population of department
Activity-Weekday-08h	Sum of all users' activities (calls/SMS) in department during weekdays in 08-09h time interval divided by population of department
Activity-Weekday-09h	Sum of all users' activities (calls/SMS) in department during weekdays in 09-10h time interval divided by population of department
Activity-Weekday-10h	Sum of all users' activities (calls/SMS) in department during weekdays in 10-11h time interval divided by population of department
Activity-Weekday-11h	Sum of all users' activities (calls/SMS) in department during weekdays in 11-12h time interval divided by population of department
Activity-Weekday-12h	Sum of all users' activities (calls/SMS) in department during weekdays in 12-13h time interval divided by population of department
Activity-Weekday-13h	Sum of all users' activities (calls/SMS) in department during weekdays in 13-14h time interval divided by population of department
Activity-Weekday-14h	Sum of all users' activities (calls/SMS) in department during weekdays in 14-15h time interval divided by population of department

Feature Name	Description
Activity-Weekday-15h	Sum of all users' activities (calls/SMS) in department during weekdays in 15-16h time interval divided by population of department
Activity-Weekday-16h	Sum of all users' activities (calls/SMS) in department during weekdays in 16-17h time interval divided by population of department
Activity-Weekday-17h	Sum of all users' activities (calls/SMS) in department during weekdays in 17-18h time interval divided by population of department
Activity-Weekday-18h	Sum of all users' activities (calls/SMS) in department during weekdays in 18-19h time interval divided by population of department
Activity-Weekday-19h	Sum of all users' activities (calls/SMS) in department during weekdays in 19-20h time interval divided by population of department
Activity-Weekday-20h	Sum of all users' activities (calls/SMS) in department during weekdays in 20-21h time interval divided by population of department
Activity-Weekday-21h	Sum of all users' activities (calls/SMS) in department during weekdays in 21-22h time interval divided by population of department
Activity-Weekday-22h	Sum of all users' activities (calls/SMS) in department during weekdays in 22-23h time interval divided by population of department
Activity-Weekday-23h	Sum of all users' activities (calls/SMS) in department during weekdays in 23-24h time interval divided by population of department
Activity-Weekend-00h*	Sum of all users' activities (calls/SMS) in department during weekends in 00-01h time interval divided by population of department
Activity-Weekend-01h*	Sum of all users' activities (calls/SMS) in department during weekends in 01-02h time interval divided by population of department
Activity-Weekend-02h*	Sum of all users' activities (calls/SMS) in department during weekends in 02-03h time interval divided by population of department
Activity-Weekend-03h	Sum of all users' activities (calls/SMS) in department during weekends in 03-04h time interval divided by population of department
Activity-Weekend-04h	Sum of all users' activities (calls/SMS) in department during weekends in 04-05h time interval divided by population of department
Activity-Weekend-05h	Sum of all users' activities (calls/SMS) in department during weekends in 05-06h time interval divided by population of department
Activity-Weekend-06h	Sum of all users' activities (calls/SMS) in department during weekends in 06-07h time interval divided by population of department
Activity-Weekend-07h	Sum of all users' activities (calls/SMS) in department during weekends in 07-08h time interval divided by population of department

Feature Name	Description
Activity-Weekend-08h	Sum of all users' activities (calls/SMS) in department during weekends in 08-09h time interval divided by population of department
Activity-Weekend-09h	Sum of all users' activities (calls/SMS) in department during weekends in 09-10h time interval divided by population of department
Activity-Weekend-10h	Sum of all users' activities (calls/SMS) in department during weekends in 10-11h time interval divided by population of department
Activity-Weekend-11h	Sum of all users' activities (calls/SMS) in department during weekends in 11-12h time interval divided by population of department
Activity-Weekend-12h	Sum of all users' activities (calls/SMS) in department during weekends in 12-13h time interval divided by population of department
Activity-Weekend-13h	Sum of all users' activities (calls/SMS) in department during weekends in 13-14h time interval divided by population of department
Activity-Weekend-14h	Sum of all users' activities (calls/SMS) in department during weekends in 14-15h time interval divided by population of department
Activity-Weekend-15h	Sum of all users' activities (calls/SMS) in department during weekends in 15-16h time interval divided by population of department
Activity-Weekend-16h	Sum of all users' activities (calls/SMS) in department during weekends in 16-17h time interval divided by population of department
Activity-Weekend-17h	Sum of all users' activities (calls/SMS) in department during weekends in 17-18h time interval divided by population of department
Activity-Weekend-18h	Sum of all users' activities (calls/SMS) in department during weekends in 18-19h time interval divided by population of department
Activity-Weekend-19h	Sum of all users' activities (calls/SMS) in department during weekends in 19-20h time interval divided by population of department
Activity-Weekend-20h	Sum of all users' activities (calls/SMS) in department during weekends in 20-21h time interval divided by population of department
Activity-Weekend-21h	Sum of all users' activities (calls/SMS) in department during weekends in 21-22h time interval divided by population of department
Activity-Weekend-22h	Sum of all users' activities (calls/SMS) in department during weekends in 22-23h time interval divided by population of department
Activity-Weekend-23h	Sum of all users' activities (calls/SMS) in department during weekends in 23-24h time interval divided by population of department

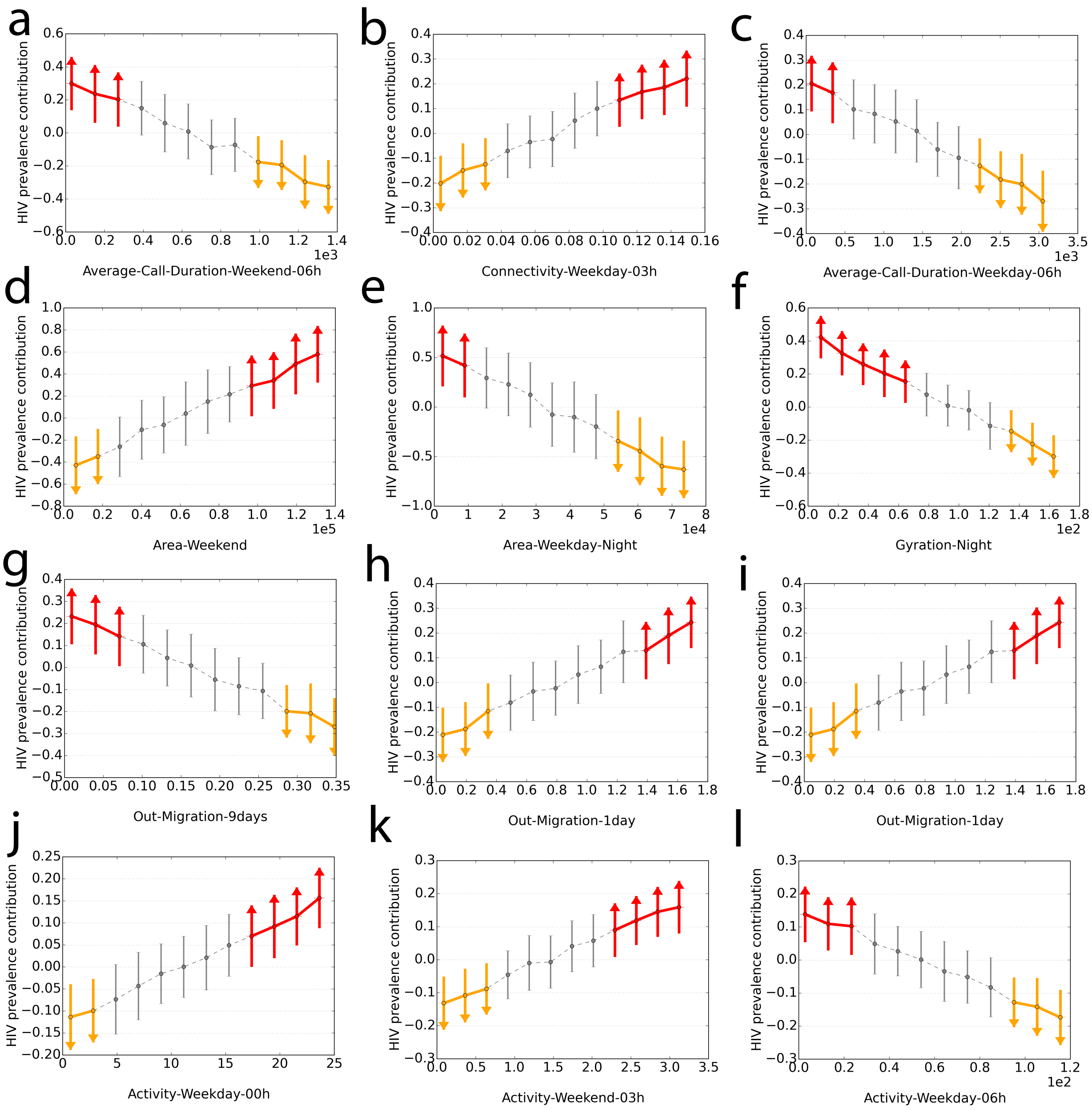

Figure S2: Feature contribution graphs for 12 features; ranked from 4th to 6th place for 4 types of features. Points correspond to mean contribution and error bars correspond to standard deviation. Red color indicates strong association to higher HIV, and orange to lower HIV prevalence.
